# Basketball in the Northland: a community account of the political economy and racial dynamics of youth basketball in a “hockey town”

**DOI:** 10.3389/fspor.2023.1250325

**Published:** 2023-11-20

**Authors:** Jason Torkelson, Tyrell Robinson

**Affiliations:** University of Minnesota - Duluth, Duluth, MN, United States

**Keywords:** basketball, culture, sports, sociology, race, class

## Abstract

This article utilizes 27 in-depth interviews to explore the socio-cultural position, surrounding racial politics, and local economy of youth basketball in the somewhat novel setting of Northland—a mid-sized Midwestern city where hockey dominates the sporting landscape, indoor basketball facilities are relatively sparse, and snow coverage that frequently lasts more than half the calendar year hampers outdoor play. Narratives are primarily gathered from basketball community members (e.g., organizers, coaches, and former players) and connect with city histories of redlining, school redistricting, and regional deindustrialization in ways that inform present-day constructions of playing style, stereotyping, race, and rivalry, as well as the current horizons of opportunity and access for Northland youth. Our ground-up community-focused data foreground efforts and hurdles to providing effective mentorship and actualizing basketball's ethos of community wellness in this fold as this transpires against the increasingly higher stakes contours of youth sports broadly, which informs pertinent issues for player development, but contrasts the common tendency toward engaging these from elite levels downward, or within newer cultures of upper-/middle-class parenting. Ultimately, the descriptively rich ways and frequent starkness with which potential challenges regarding player interest, coaching, funding, and access—both in and out of the recent COVID-19 restrictions—come to bear through our grassroots approach at the unique intersections of Northland's weather, sports, and racial–political climates may clarify connections with similar issues elsewhere.

“One of the reasons you see a lot of low-income people playing basketball is because it's cheap—you [just] need a pair of shoes and one of you needs a basketball … I couldn't have afforded to play hockey [growing up] … just because we were poor.”

(Evan—48, Social Worker, Northland basketball community member)

“In the [Northland] winter, it's [a] tough lifestyle because you're stuck inside all the time. It gets dark early; it's cold; there's not that many opportunities to go to the gym and do anything. Hockey, it's like every ice rink, indoor, outdoor, frozen pond, kids are playing. Basketball it's … the complete opposite … There's no gym availability.”

(Berndt—26, former Division 1 collegiate basketball player from Northland)

“I can't stand the lack of gym space in this area. COVID made that worse … These kids are losing their minds.”

(Daran—30, basketball organizer and former professional player from Northland)

## Introduction

“All you need is a hoop, shoes, and ball” is a common refrain among basketball enthusiasts, as here reflected by 48-year-old Social Worker Evan. The sport has indeed long stood out in the American public imagination for its accessibility and potential connection with under-resourced populations. Surrounding mediated imageries—frequently deliberately racialized—of aspiration and the tapestries of urban life seethe through Nike marketing campaigns (1), the National Basketball Association's public relations interface (2), and prominent films like 1994’s *Hoop Dreams* detailing two young African American Chicago males questing for a better life through basketball. Significant policy initiatives have also been threaded in this fold. For example, the “Midnight Basketball” campaigns of the 1990s garnered bipartisan support via the claims that basketball can simultaneously springboard social development and integration for the underserved while providing a cost-effective mechanism for controlling perceived troublesome or “at-risk” populations (3).

Social scientists have voluminously underscored the racial and socio-economic significance of basketball (3–11), community relations and cultures surrounding basketball in places such as urban parks (12–15), and the role engagement with basketball—both formally and informally organized—may play in growth and development (13) [see also (16)]. However, what about these focal points when the foundational association between basketball and accessibility in cities cannot as readily be taken—e.g., where outdoor play is significantly limited by weather and indoor basketball facilities may additionally be relatively sparse?

In this paper, we approach these—and other related—issues while telling the story of youth basketball in the predominantly white, mid-sized Midwestern city of Northland, where among the coldest average temperatures and highest snowfall rates in the contiguous United States frequently finds outdoor snow coverage lasting more than half the calendar year. In contrast to the depictions of dribbling down the street, public park pickup games, and outdoor practice collected from the—relatively temperate—setting of even Chicago for *Hoop Dreams*, as 26-year-old homegrown former Division 1 collegiate basketball player Berndt notes, outdoor play in Northland is difficult, if not impossible, for long periods. Likewise, in Northland, AAU (Amateur Athletic Union) teams are presently limited by space and current reputation, and there is no “blue-blood” high school basketball program—increasingly essential ingredients for success in the “higher stakes” 21st Century culture of youth sports—of the caliber the young men from *Hoop Dreams* pursued transfer to that can more maximally showcase and cultivate local talent. In all these veins, the comments of both Berndt and Evan further connect with the great extent to which Northland's sports culture, identity and facilities funding have come to be dominated by hockey in recent decades.

Berndt and Evan's narratives are among 27 interviews collected primarily from sports organizers, coaches, and former homegrown players for a larger documentary oral history project on basketball in this novel location. These jointly color local constructions of race, rivalry, and contours of player development in dialog with Northland's history, socio-demographic layout, politics of facilities and school funding, as well as basketball's position relative to hockey in the consciousness of this whiter and colder region. Furthermore, as Daran's above appraisal of Northland basketball facilities hints, data are collected at a timely moment to begin considering possible aftereffects of the COVID-19 restrictions of 2020–2021, especially since these might be uniquely inflected in this locality.

Like many sociological questions, vital to best informing current moments is a connection with deeper history (17). Indeed, the current state of youth Northland basketball cannot be fully told in the absence of attendant histories of redlining, school districting, and regional deindustrialization, all of which have culminated in the city's fairly socio-economically and racially segregated player pipelines of today. In the primary spirit with which our data were collected, we use our interviewees’ narratives to tell this history, and emphasize standpoints from within the Northland basketball community to tell their story of the present-day Northland sporting landscape as play resumes post-COVID-19 restrictions. Our ground-up community-based data highlight issues important to understanding basketball (and sport generally) regarding the dynamics of sport and community, youth development, political economy of youth sports, race, and history and place, among others. Here, the potential starkness of how Northland's unique weather, sport, and racial–political climates have historically collided may clarify approaching similar issues elsewhere, particularly concerning how central questions of access and in-season facilities—already longstanding to Northland basketball—may loom larger in coming years generally insofar as certain strands of youth sports are becoming more rigidly organized, high-stakes, and potentially requiring more corresponding year-round formal accommodations.

## The racial significance of (youth) basketball in America and American cities

Basketball in America has always had a relationship with weather and the intersections of socio-economic status and race, albeit one containing shifts. This goes back to the sport's 1890s foundations when James Naismith expressly developed basketball as a means of providing indoor exercise during harsh Massachusetts winters. Basketball quickly expanded through the Northeast but especially found a home among poorer immigrant populations whose “children … sought to shed the lifestyles of their parents and embrace the new country” (18). Basketball not only provided an inexpensive means but was also seen by youngsters as a path out of the early 20th-century urban ghettos when collegiate and professional teams were formed (19). Many of the first formal teams were segregated in accord with racial and then-significant boundaries around Euroethnic groups (e.g., Jews, Germans, and Irish). The first predominant face of basketball, however, if any, was Jewish (18, 20). By the 1930s, racialized stereotypes were firmly connected with the playing style, where Jewish players were perceived to possess natural synchronicity with valued skill sets of the time. For example, the *New York Daily News* lauded Jewish players’ “alert, scheming mind, flashy trickiness, artful dodging, and general smart aleckness” [cited in (19)].

This is a far cry from the sport's present state, where Jewish players of prominence are rare and, more significantly, basketball is now primarily marked as “black” in ways that can anchor African American identities, esthetics, and systems of meaning-making (11, 21) [see also (22)]. To be sure, there were African American teams, or “black fives,” in the earlier 20th century, which existed alongside the publicly prominent Harlem Globetrotters until the NBA was formally integrated in 1950. However, by possessing generations of history with America, earlier-century black youths were not in the process of acculturating to America's sporting tradition as were arriving European immigrants (23). African American youth had been longer situated in a culture dominated by baseball, and baseball anchored collective identity and modes of expression among black youngsters until at least the 1950s (24, 25).

A substantial part of the change stems from the fact that much basketball came to be played outdoors as basketball courts were placed in urban public parks [see also (26)]. These made play on regulation hoops—even full courts—readily available to anyone with shoes and a ball. Informally structured pickup game cultures and what has since been branded as “streetball” developed, introducing a faster, freewheeling mode of play that contrasted the mid-century white vanguard. At the intersections of race in urban contexts, which can include violence, underfunded schools, and low opportunity for mobility, these processes may in ways reflect what Hunter and colleagues (22) had more broadly labeled as “black placemaking,” or the process of “shift[ing] otherwise oppressive geographies of a city to provide sites of play, pleasure, celebration, and politics” (p. 34). As Runstedtler (27) more specifically labels it, “black ball” seeped into and assumed the dominant face of the professional ranks as the NBA lifted its racial quota system and merged with the more black ABA in 1976. By the end of the 1970s, black players were perceived to have “imported their aggressive, flashy, individualistic, and above-the-rim style from the playgrounds of black neighborhoods to the pro-game” (p. 2).

If there was a racial tipping point for the sport's racial associations, it indeed came through the 1970s when the NBA went from being roughly half black at its start to 80% at its end, all transpiring in the wake of Civil Rights and the 1960s athlete protests [cf. (28)]. Perhaps, in contrast to the degrees of safer racial voyeurism arguably constructed since then, the reception of the sport's changing face and playing style was especially fraught with white owners, fans, and sportswriters (27). The NBA—and much higher-profile basketball generally—struggled with white fan interest through the entirety of the 70s. Black players were frequently treated as social problems on and off the court (e.g., inherently undisciplined). They were perceived as contaminating Naismith's game with America's urban crisis. New corresponding racial stereotypes also emerged—from the developing white gaze, “black players were so skilled … that white sportswriters and fans mistook their seemingly effortless virtuosity for a lack of effort” (27), p. 246 [see also (29)].

These stereotypes undergird critical concepts such as urban ghettocentrism, or the essentialized black athlete, which itself emerged with modern sports (5, 6, 30). Aspirations of upward mobility in this fold may be fashioned through basketball in new racialized manners, particularly as deindustrialization and legally sanctioned practices of redlining into the 1970s rendered the idea of the suburban dream, more or less the American Dream, a figment of imagination for blacks in urban cities (5). At the same time, substantial material benefit was lent to and “whitened” populations such as Jews and other Euroethnic groups [see (31–33)] that had before been more uniquely stereotyped in the play of basketball.

Race then certainly continues to be a focal point in the play and cultural resonance of basketball. For youth, access and participation in sports vary significantly by race, and kids of color can be ushered toward some sporting activities based on racial attributions (8). Racial stereotyping of players and play itself remains pervasive and well documented in the 21st century in accord with concepts like “white ball” vs. “black ball” (4, 7, 34). As ethnographies by Buford May (35) and Brooks (29) on elite high school basketball indicate, this can now play a role in conditioning recruitment prospects to the collegiate ranks and beyond. Essentialist stereotypes can mask player work ethic, and attributions of black-coded playing style can strike discord with perceptions of suitability for higher levels of play.

Further in this vein, the sort of pipelining required for successful collegiate and professional play has itself potentially shifted in recent years in ways that may be forming new types of social closures in the 21st century. Divestments in public parks and sports generally across America followed the 2008 market crash. This has helped pave the way for an ascendant private model of youth sports in which “higher stakes” formal play (over fun) often requiring substantial travel team and personal training expenditures are normalized—if not required for keeping the developmental pace—for even elementary-aged children (see Flanagan, 2022). This youth sports industry is mushrooming so fast that one prominent report from Wintergreen Research Inc. not only pegged its growth at roughly 90% since 2010 but also forecasted it to grow from roughly 37 to 65 billion dollars annually from 2021 to 2028 (36). For critics like Coakley (37), this privatization operates flush with corresponding neoliberal social logics that emphasize individualism over community and emergent cultures among wealthier families where the worth of parentage is appraised via the relative successes of one's children [see also (38, 39)] over those who may be “priced out.”

Regarding effects among basketball participants (formally organized or ostensibly informal), Viyera's (12) foray into Philadelphia pickup games shows play serving as a center for galvanizing community and support systems. Complementarily, for DeLand (14, 15), the social accomplishment of games is an active process of acclimating to and deploying repertoires of conflict management, where skills of handling disputes and building credibility are learned. The ways sports can have a lasting impact on lives and foot social development through mentorship figures, such as coaches, are starkly reflected in the literature on coaching (16, 40). Generally, some authors, like Hollander (13), have even posited engagement with basketball—which requires, among other things, one to handle rapidly changing situations while delicately balancing pertinent questions such as individuality/collectivity—to be an ideal socialization medium for the ways it mirrors and may prepare individuals for engaging issues of fairness, leadership, and equality central to late modern societies.

## Investigative approach, data, and methods

We investigate these reviewed issues on a grassroots level in the relatively unique locality of Northland as told primarily by members of its somewhat small basketball community. Relations to basketball in our final sample include key organizers, sport and school administrators, coaches, figures in local politics, community workers, and homegrown players with national and international profiles who retain ties to home. All of these interviewees identified with and crucially voiced passion for and/or investment in Northland youth, basketball, and/or player development.

Interviews were semi-structured but aimed to connect with the state of Northland basketball for player development, opportunity, community, facilities, economics, and the general tenor of the sport as it grapples with the contemporary youth sporting landscape situated in this whiter, colder region relative to other sports like hockey. Data were notably gathered in conjunction with a documentary oral history project on Northland basketball, the spirit of which is reflected in our presenting Northland basketball's story below. Further here, additional interviews were conducted with individuals more outside the Northland basketball community, where their experiences and expertise about the racial, schooling, facilities funding, and socio-economic histories of Northland might complement or complicate our core informants’ perceptions of Northland basketball. A total of 27 formal interviews were conducted, with 23 being face-to-face and four over Zoom. While the picture these narratives collectively paint are, by method, not necessarily reflective of any objective condition of things in Northland, they do importantly foreground a particular community's perceptions in ways that can valuably contribute to how an array of key issues surrounding basketball and youth sports are understood—e.g., its political economy, local geography, youth development, social inequality, understandings and experiences of race and racism, local history, collective memory, consequences of public divestment, and sport as a potential tool of community.

Word-of-mouth and snowball sampling methods were deployed. While this risks perspectives remaining confined to one network (41), pitfalls may be offset by several different interview chains yielding responses, the smaller size of Northland basketball itself, and the diversity of relationships contained in our sample (see Table 1). Interview durations ranged from 25 to 90 min. The youngest interviewee was aged 21 years, and the oldest was 73. In all, the variety of interview chains and positions around Northland basketball validated common themes.

**Table 1:** Informant details.

Name	Int age	Race	Gender ID	Profile
Evan Botten	48	White	M	Social worker, Middlehill player
Quincy Wright	31	Black	M	Food service, regional player, Middlehill alum
Kori Huttunen	30	White	M	Teacher, former D3 player, coaching experience
Martin Gribelin	23	Black/white	M	Food service, former Lakecrest player
Bruno Ernst	21	White	M	National guard, former regional player, coach, community volunteer
Calvon Cooper	56	Black	M	Human Rights Officer, former D3 player, coaching experience
Kendra Wheeler	40	White	W	Northland city planner
Daran Sampson	30	Black/white	M	AAU organizer, former Lakecrest, D1, professional player
Arvid Stroh	27	White	M	Regional player, D1 player, basketball academy organizer
Irina Cronenberg	39	White	W	Youth director, former JUCO player
Daniel Netze	48	White	M	Former prominent Northland politician, Middlehill player
Helga Hinrichs	55	White	W	Freelance writer, local social activist
Katy Abbott	41	White	W	Nurse, former AAU coach, Douglaston coach
Parker Adams	71	Black	M	Gym trainer, community volunteer
Elias Rosin	48	White	M	Former Middlehill player, Douglaston coach
Berndt Buras	26	White	M	Regional player, D1 player, basketball academy organizer
Tommy Lockhart	34	White	M	Community college instructor, coach
Nick Gillian	47	White	M	School administrator, Lakecrest alum, sports organizer
Cameron Korpela	73	White	M	Retired teacher/coach, basketball community member
Tina Scott	55	Black	W	School administrator
Katja Manninen	49	White	W	Basketball director, former Middlehill player
Tony Pelzer	55	White	M	Douglaston athletic director, former Middlehill and D2 player
Janusz Broda	30	White	M	Store manager, regional player, Middlehill
Roman Jost	66	White	M	Professional, former Lakecrest and D1 player
Kathrine Schubert	60	White	W	Former coach, athletic director, Middlehill and D3 player
Enrique Lukes	44	White	M	Youth counselor, former Douglaston player and coach
Qualeek Gibbs	27	Black/white	M	Group home worker, former Douglaston player

Interviews were transcribed and coded, and strong intercoder reliability was found. Perhaps, as the structure of the findings shows, most central of all are the histories of three core city public high schools in Northland, which our informants overwhelmingly connected to today's fairly racially and socio-economically distinct sections of the city in ways they frequently believed conditioned opportunity and play. These are the relatively poorer “Douglaston High,” the more affluent and whiter “Lakecrest High,” and the relatively mixed but now shuttered “Middlehill High.” The interviewee names and locations are pseudonymous.

## The climates of youth Northland basketball

### Weather

The larger, predominantly white Northland metro region centers around a mid-sized port city that has connected the area's industries to the world since the mid-19th century. Northland winters can be particularly, if not notoriously, harsh. The average temperatures consistently rank among the 3–5 lowest among cities in the contiguous US. The annual snowfall rates likewise reside comfortably in the top 10 for American cities. Northland's proximity to a major lake makes for colder fall and spring seasons that frequently find ground snow coverage lasting more than half the calendar year.

### Sports

It is perhaps no surprise that this indoor sport would find a place in a weather climate like Northland's when basketball proliferated through earlier 20th century America. Organized Northland youth basketball goes back to at least the 1910s when programs were instituted at the local city high school of Middlehill and soon thereafter at cross-town Douglaston High upon its founding. Regional high school basketball grew tremendously toward and past mid-century (42) alongside the city's soaring population and then-thriving industry. Emblematic of this was the introduction of another city public high school and team, Lakecrest, the same year the NBA's first black players signed, and in the wake of a Douglaston state championship that galvanized the city just 3 years prior. As Swenson [(42), p. 89–90] characterized the scene following Douglaston's 1947 title game triumph in a large metropolis south of town:

The … Mayor … was informed that the [Douglaston] team would be arriving back … via three private automobiles. This set in motion plans for an impromptu welcome home celebration! Sound trucks from KDAL radio and police vehicles went up and down the streets … informing fans when the team would arrive. The result was a fourteen mile line of cheering fans.

If there were halcyon days for high school youth basketball in Northland's sports climate, they arguably came in the wake of Douglaston's championship through the decades following the Second World War. Cross-town rivalries swelled and captured attention, especially through the 1970s, a decade in which Middlehill won the city's only other top-class boys state championships to date. Many of our older respondents reflected on this fervor. Tony, a 55-year-old Middlehill alum and current Douglaston sports administrator, fondly recounted, “Lakecrest and Middlehill would play regular season games at [the college gym] because they didn't have enough seats … They even played a regular season game at the old downtown arena because those games in the 70s would attract so many people!” Elias, who is 48 years old and likewise came through Middlehill, subsequently played collegiately, and now works with Douglaston athletics, importantly connected some of the underlying animus for these rivalries to socio-economic disparities between Lakecrest High versus others when telling us “People would come out for games when the Northland schools would play each other. I think it was always understood at least by Middlehill kids and Douglaston kids that Lakecrest was where the money was … There was … a little bit of a further rivalry, animosity.” Here, Nick, a 47-year-old lifetime Northland resident and school administrator, more pointedly placed Northland's status as a “basketball town” in the past:

There were some crazy rivalries between all three of the high schools and the gyms would sell out. The downtown arena would fill up … There really was just a lot of excitement around basketball. It was, in a lot of ways, a really good basketball town … It really brought a lot of people together around basketball … It was just a fun place to be.

Basketball, and much of life in Northland broadly, has since been set in more challenging times. Deindustrialization hit hard when a prominent steel mill closed in the 1970s. The population subsequently thinned rapidly through the 1980s. This all was to such a point the city ranked in the top 10 most recession-ridden metropolitan areas nationally, which led the then-mayor to ask the Reagan administration for “programs and solutions which will help our unemployed citizens survive this winter” (43). Perhaps, the scene around the main freeway in the 1980s could not have more starkly contrasted the hero's welcome the Douglaston championship team received off its in-bound path four decades before with a large out-bound billboard reading, “Will the last one leaving [Northland] please turn out the light.” The population stabilized in the 1990s but not without the city poverty rate climbing to 15%, which then peaked above 20% following 2008's market crash.

Youth basketball has weathered these years, but not as well as hockey. If it ever did not before, hockey now resoundingly assumes the mantle of the region's sports identity. With almost no exceptions, present perceptions of Northland basketball were bound by its holding secondary status to hockey. Arvid, a 27-year-old former Division 1 player and current youth basketball director, said of growing up as a basketballer in Northland that “hockey's probably the major sport that most kids play in the winter, just being in the cold and everything like that, so it's a little different [here].” Former Douglaston player, 27-year-old Qualeek, adds, “Hockey is the biggest thing around here … Hockey players are a lot more noted around here, just because there's a ton of players that are in the NHL now that are from here. So that's a big thing.” Turning the focus to development, Bruno, a 21-year-old youth basketball volunteer, bluntly said, “Basketball development in Northland has been a joke … it's a hockey community.” Further on this front, Berndt underscored what he perceived to be a pronounced need to find proper mentorship and community in Northland's sporting climate when reflecting on what footed his ascension to collegiate play: “It['s] a hockey city. It's a hockey state. Everything is about hockey. So I wouldn't say Northland was great for our basketball preparation or to get us going, but we were fortunate enough to meet some people … But besides that, it wasn't the greatest.”

Regarding the dominance of hockey, the extent to which a heralded Division 1 college hockey program looms over all Northland sports perhaps cannot be overstated. The college teams (men's and women's) now play in a new 2010 arena partly built with public funds. The men's team specifically routinely develops NHL prospects, and they now undoubtedly comprise the hottest sports ticket in town. Tony, a lifetime resident, accounted for changes he saw in the city's media and sports focus:

As a basketball guy … what's the headline story all the time? It's [college team] hockey … There used to be articles like this written in the newspaper about basketball games. Now that's a whole other topic of what media can afford, the sustainability of media, but there used to be a feature basketball game twice a week where they'd write a big story.

Powerhouse high school hockey programs likewise command a substantial presence. These often recruit players external to the region from families who possess resources to relocate their children. None are more prominent than hockey “blue-blood” Harris Ridge High, adjacent to Northland city, and a focal point for comparisons of regional hockey versus basketball player pipelines among several. However, 48-year-old former politician and Middlehill player Daniel perhaps best described perceived disparities between hockey and basketball opportunities in Northland here:

If the parents are like … “Hey, we are a hockey family … we want our kids to play hockey, we love hockey … It's time for us to buy a house. Where are we going to buy our house?” Well, you're gonna buy your house in Harris Ridge … And if they plug in at Harris Ridge, they're going to have these … hockey facilities … If you know that you want your kids to play basketball, where do you buy your house? In our region? … I don't know if there's that answer right now.

For basketball aspirants to showcase skills at levels similar to what Northland hockey players could potentially enjoy in-region, many believed relocation or travel to the Metropolis well south of Northland was currently required. Bruno describes this dynamic “No one's really coming to Northland … because … it's hard to get noticed up here … A lot of kids would have to travel down to the Metropolis for an AAU team … [to] be able to have someone see them.” Huntley High, the closest “blue-blood” high school program to Northland, as some mentioned, resided in the suburbs of the Metropolis. Of how their team assembly differs from anything in Northland, former Division 1 player and current youth basketball director Daran described Huntley as “taking bigger kids … Huntley has all Black kids on their team/ … /It's one of the wealthiest suburbs in [the Metropolis]! There ain't fucking 10 Black kids and those families [now] all live in Huntley and play on their team … [There is a] hoarding of players at Huntley!”

Recent data from “Project Play” show hockey parents reporting expenditures averaging more than six times their basketball counterparts (44). However, given the differential racial associations between hockey and basketball [(45); see also (46)] that have shaped since the 1970s in particular (27), it may not actually be surprising that the white-coded sport of hockey came to dominate this largely white region, despite the economic tribulations experienced through these times.

Regarding whiteness, engaging with basketball in Northland and experiencing its local sports hierarchy from basketball's position notably found many among our white respondents actively racializing cultural whiteness, a process otherwise generally documented as difficult for whites, if not even potentially hampered by fundamentally embedded “ignorance” [cf. (47); on white young persons in America specifically here, see also (48, 49)]. Our data here may specify a local condition where whiteness as a socio-cultural location or set of practices comes to register on the consciousness of whites, rather than more comprehensively operate as the blank or unnoticed force it has conventionally otherwise been theorized [for a recent review, see (50)].

Very basically, Tony professed: “Basketball opened my eyes to race.” Elias directly connected with interracial exposure he believed basketball specifically offered in the whiter Northland:

I noticed it [race] through basketball, really. My interactions with African American kids, or other ethnicities, Native American, etc., had been pretty minimal during my elementary school years. But then as I went into junior high and started to play basketball … I mixed the most with kids that were of different race than me.

Janusz, a 30-year-old lifelong white resident, similar to others asserted, “Hockey players in Northland are treated like the lead singer of the band and the other athletes are the bass player, the drummer,” and also told us that “in my experience, growing up here, people favored hockey because … there's more White people here.” Furthermore, he said of his understandings:

I was a White basketball player, and in [this state] that is less common than elsewhere … It turned out to be one of the best things that ever happened to me. Honestly, I understood race relations better than everybody around me at a really early age. I have a lot of friends from very, very different walks of life than me because of it. And I think I did a good job of you know, showing … that not all [Northland] White people are hockey families.

For one more example, Evan perhaps most pointedly attributed his experiences with the differential racial composition of Northland basketball to his cultivating awareness of white normativity and how racial minorities are situated:

I played a lot of places where I was the only White guy … It's like, I'm the outsider here … I also kind of realized, it's like, I have the entitlement of being White. I could leave that little scenario anytime I wanted, and go back to the rest of the world where I'm the norm … Some of my friends who are black, it's like, that was the only time they … didn't have to worry about … being treated like an outsider and … [be] treated different because of the color of their skin … What they had to deal with … it's kind of just that … moment of like “Shit, this would suck if I did the time.”

### Race

The racial climate of Northland possesses a history as deep and frigid as the lake waters it is nestled against. Initially colonized by groups of Northern European descent, African Americans first sizably moved to the region beginning in 1915 upon being recruited for steelwork due to their comrpising a relatively cheap labor force. Only 5 years later, three black men were victims of a now-notorious lynching spurred by false rape charges. “Thousands of people lined the streets and watched” as a mob stormed the jail where the accused men were held; aftermath photographs were taken featuring white citizens posing next to the men's bodies; some of these images were even later featured on postcards (51). The region's racial climate was so chilled that many Blacks left throughout the 1920's for fear of further lynchings (51).

The lynching can still cast a pall over Northland's race relations, if not via operating as its “elephant in the room” [cf. (52)], as an occasional explicit reference point or its embodiment in America's first public lynching memorial that now resides in the city's downtown (53). Some unsurprisingly made unprompted references when describing Northland's current racial dynamics. However, none were more poignant for basketball than the anecdote of 56-year-old basketball organizer and Human Rights Officer, Calvon. He described black players from a Middlehill team recognizing some of their teammates’ relatives posing in one of the lynching photos when it appeared on the cover of the newly released book on the incident. Middlehill just defeated rival Lakecrest to earn a spot in the state tournament, but the book cover's revelation almost prompted the black players to forfeit rather than pursue a championship with their white teammates:

There was actually a [Middlehill] basketball team that was going to state until the cover of that book came out … The Black athletes recognized some of their Italian teammates’ relatives. And it actually took a long conversation before they went to state because the question was, “Why were your grandparents there at a lynching?” And it was a really hard conversation that took place in a very short amount of time.

### Neighborhood and school districting

Much of the post-lynching structure of race in Northland—including the time when basketball picked up steam through at least the 1970s—meshes with an unfortunate but familiar trajectory in American cities of redlining, neighborhood racial covenants, and ultimately the differential distribution of benefits like the GI Bill that birthed many of the American suburbs and cemented degrees of lasting racial segregation. Northland was indeed appraised by the Home Owners’ Loan Corporation in the 1930s–1940s, the fallout of which continues to produce compounding consequences into the 21st century generally (54).

Here, the fuller backcloth against which organized youth basketball transpires and conditions attendant aspiration, opportunity, and play in Northland from the high school level downward is not complete without one additional, interweaving, and very significant thread, which is (the politics and socio-demographics surrounding) the eventual closing of Middlehill High. With a heavily winnowed population and cratered 1990s economy, the city looked into possible school reconfiguration plans, just one of which involved permanently closing Middlehill. Perhaps akin to school closure decisions elsewhere that have been documented with respect to their relation to local housing policy histories and attendant racial dynamics (55), Middlehill was geographically situated between Lakecrest and Douglaston, and it was the most socio-economically and racially mixed. As Janusz, a member of among its final classes, recollects, “It was kind of just like this big melting pot.” Middlehill was also significant for its reputation as a—if not to some, being “the”—basketball school for much of the time it stood. Emblematic are the almost identical responses of Daniel and Tony on city school sports reputations. Tony said, “Middlehill was always a basketball school. Lakecrest was always a hockey school. Douglaston was always a football school.” Likewise, to Daniel, “Lakecrest was a hockey school. Douglaston was the football school. Middlehill was the basketball school.”

The decision to move forward with the specific plan to shutter Middlehill was handed down by the school board in a swift—and according to many, scandalous—act cloaked by city basketball. Bruno attests, “They voted to close it during a Middlehill versus Lakecrest … game, because there's no parents there … It wasn't supposed to be on the agenda. They like threw it up and said we're closing Middlehill, and it got such backlash.” Daniel played in the game Bruno notes. Here, he describes his disappointment encountering the news in the glow of just beating Lakecrest in a significant step toward a state tournament berth to be so jarring he partly attributes it to shaping his path to politics,

[I] wake up the next morning thinking “This is going to be the best day of my high school career” because we just beat the rivals. We're one game away from state and this is going to be awesome. I went downstairs to read the newspaper … I read the first paragraph and it said, ironically, “On the same day that the school board voted to close Middlehill as a high school” … And I had to read it again like what just happened?/ … /It was also when I started getting politically involved because I saw like, “Hey, here's this kind of movement of what you can do to kind of make a case and you can change policy” because then the school board reversed its decision and kept Middlehill open as high school.

The school board indeed initially turned back given the blowback received, but Middlehill eventually closed in the wake of the 2008 financial crash 1 year after the new downtown college hockey arena opened. This has resulted in a new landscape for organized youth sports in terms of the city schools. Tina, the 55-year-old former school administrator we interviewed about school sports funding, facilities, and management, said of the closure's implications that “Middlehill was the more … racially diverse school … And in … socio economics, they had a wide range of people … [It] create[d] a ‘haves and have nots’ system with going to two high schools.” Reflecting on now working within the post-Middlehill landscape, the 49-year-old youth basketball director Katja asserted, “Closing Middlehill … just really made the disparity so apparent to everyone in town … No one can deny it now.” However, 40-year-old Kendra, the local historian and urban planner we spoke to, best connected these histories across Middlehill's closure:

When you look at some of the redlining maps in Northland, we [now] have the two high schools for the … the public school district … Douglaston on [one] side of Northland and Lakecrest on the [other] side of Northland. And the demographics of those two different boundaries for those schools are very stark … [The] percentage or share of people of color who live in the Douglaston side of Northland is much higher than in the Lakecrest side of Northland. So right there we have in our high schools, this significant racial demographic divide, and it stems from the way that the government decided to invest in or not invest in certain neighborhoods … A lot more of the Douglaston neighborhoods were given those much lower grades on the redlining maps … There's a lot more concentration of people with lower incomes and a lot more concentration of people of color than on the other side of town.

The new configuration of Northland's city high schools then finds the area's deeply embedded racial and socio-economic histories culminating in two fairly divergent player pipelines that now shape play and aspiration in the shadows of hockey, a uniquely unforgiving weather climate, and in the absence of what some considered the region's most noteworthy basketball school from recent decades. How this may condition the local construction of play, race, community, facilities access, and shape mentorship and coaching—into and out of the COVID-19 pandemic restrictions—is to where we next turn.

## Two sides of the tracks: racialized pipelines and play

The redistricting was central to understandings of current youth local basketball. At its most basic level, as 31-year-old Quincy observes, “Nowadays [a] Lakecrest versus Douglaston … rivalry kind of accumulated.” Martin, a 23-year-old recent Lakecrest player, plainly characterized it as “it's hot, man. It's dope.” Another former collegiate player and Nothlander who now teaches at an elementary school spanning the border of the two districts, 30-year-old Kori, reflected on Lakecrest versus Douglaston's current potential intensity:

Lakecrest is the richest school per capita, and Douglaston is probably the poorest … There's a major rivalry. They hate each other. They always will hate each other, and there's been historically violence towards each other … There's a rift between the separation of Middlehill High School. When that happened it kind of all separated/ … /I think it's largely due to money. And I think that the issue is the opportunities provided for the Lakecrest students.

In line with Kori's comment, almost all perceived disparities across the schools’ basketball programs. Bruno says, “I do believe Lakecrest has a nicer ran program … Being able to … afford to live in … that side of town would affect how you play basketball.” The former Douglaston player Qualeek accedes, “Lakecrest is really probably ahead of everybody around here.” Elias, a current Douglaston coach, also strikingly acknowledged possible disadvantages: “Where you live in town affects … your opportunities. Right now, in terms of basketball opportunity for a kid, whatever their race is, growing up in Douglaston, the program's just underdeveloped in comparison to the program in the Lakecrest end.”

For those who played Northland city basketball, strong symbolic identity and cultural boundary work [cf. (56)] frequently emerged. This was mainly fashioned around—the inside and outside of—Lakecrest's relative prosperity. Many notably characterized Lakecrest culture and basketball as “cake eaters.” Former Middlehill player Tony discussed the “cake eater mentality of Lakecrest High School.” Irina, a 39-year-old youth basketball director, told us “the cake eaters” were “Lakecrest … where the modern or wealthy middle class live.” Even the former Lakecrester Daran owned the moniker when saying “that bougie cake eater mentality, it's like it's built there for a reason … I told people when I went there, it's damn near a prep school!”

These boundaries significantly ordered how players reported orienting themselves in Northland's organizational pipelines, engaged (versus not) opportunity, and how play itself was constructed. Regarding orientations, sticking with Daran, he not only stated that as a Lakecrester, he “had so many more resources, better coaching … more support … that's why … [Lakecrest] basketball was just better off,” but he also denied Douglaston players as being able to have pride in their school due to their underserved status:

Now that Middlehill got taken out of the mix … it's [the rivalry] a cultural thing. It's not really a pride in where you're from because, to be honest, like, there's a lot of things [about] Douglaston that don't give those kids a lot of pride … So it's a rivalry, but it's more of its cultural base. It's not based necessarily on pride in your school.

For an illustrative example of the potential strength of boundaries around Lakecrest from the outside, Middlehill alum and youth basketball director Katja illuminatingly described her team playing senior year in a condition of near mutiny because a former Lakecrest assistant became their head coach:

They hired the assistant coach from Lakecrest … The fact that they brought in someone from Lakecrest was just like … “I don't care what she has to say, she's got nothing I need to hear.” … “Why would you bring someone from Lakecrest?” And so us whole group of girls still stuck together. We still played the season, but … because she was from Lakecrest … I didn't want anything to do with it. I d[id]n't want to play.

Insofar as programmatic disparities do exist, for Douglaston district players, such boundary work can be consequential regarding whether opportunities to take on what Lakecrest might offer are engaged (versus not), and from there, experienced. What this means is Northland schools have an “open enrollment” policy, where any student can technically enroll in—and play for—either school, regardless of residence in the city. This was generally seen as a one-way street. As 55-year-old local writer Helga puts it, “There are strong class-based differences. Most parents will say I would never send my kids [to] Douglaston. They think Douglaston is full of fights, and especially Black kids are causing fights.” In echoing these appraisals, Quincy additionally touches upon ways Douglaston area kids may be negatively received at Lakecrest: “I know people that want to go to Lakecrest who live out [in Douglaston], and people don't want to go to Douglaston that live out [in Lakecrest] … Because of that district, that line … that's kind of like putting boundaries on things like ‘oh, if you don't live in this upper class area, you can't come to our school.’” Relaying similar perceptions, Evan additionally illustrates how boundary work around Lakecrest may lead some youngsters to shy away from transfer when recollecting his own orientations as a former Middlehill player:

When I was in high school … I wasn't going to fucking Lakecrest. Like that ain't happening. They had open enrollment, or whatever they called it … [Nowadays] you take in … [Douglaston area] kids, and you're putting them out [in] Lakecrest, and they don't want kids out there [Lakecrest] to want them. So you've created like this division already, how the city is set up … [T]hey [Lakecrest] still feel like this is our school you're coming to/ … /The [Douglaston] kids are still treated as outsiders.

We were told some transfers who make the leap can thrive at Lakecrest (in their athletic programs, academically, and generally), while others faced difficulties common to acclimating to whiter, more affluent environments, wherever applicable [cf. (57)]. Regarding the latter, from regional school administrator Nick's experiences, “We had students that … would transfer to Lakecrest because of maybe opportunities that they might not have had … Race played a factor … I spoke with a lot of kids to where they also felt very isolated/ … /It was a different world for them.” Regarding consequences for basketball more specifically, 40-year-old former coach and Douglaston player Enrique poignantly laid out playing time risks for Douglaston district players if they transferred but did not acculturate to Lakecrest life: “Once you complete your freshman year, you are bound to that school you choose to attend … If I open enroll at Lakecrest … I have to spend the rest of my time there. If I choose to transfer [back], I sacrifice one season of varsity eligibility.”

Further on this divide, the jerseys players came to wear—whichever way—significantly seemed to color play in accord with school reputations and cross-town stereotypes. Although the extent to which coaching or program conditions were at the root cannot be fully clear, this was generally classed and racialized across common binaries of what constitutes “black ball” versus “white ball” and ostensibly “proper” modalities of play—e.g., “disciplined,” “team-oriented,” “practiced,” versus “individualistic,” “athletic,” “extemporaneous,” as well as learning to play in the “streets” versus learning in “formal” organized or coached settings (4, 7, 27, 34).

Nick invoked class and talent differences when saying, “Lakecrest was maybe more of a finesse, just very graceful, very smooth … Douglaston really had that … blue collar mentality that they were gonna really work hard, but just never really had the level of talent as Middlehill or Lakecrest.” Similarly, Bruno on game speed and athletics: “Douglaston, it's more about ‘get the ball, go’ … There are a lot of a lot of athletes on that team … They're all about kind of just getting going, fast breaking, playing fast … Lakecrest had a lot of success with the ‘slow it down’ [style].”

Some, like Calvon, characterized these appraisals as reflective of racialized stereotypes that can negatively affect black players (5, 7, 10, 27, 29) when remarking “I think that there's always been a difference … with the Lakecrest side being considered more pragmatic, and the Douglaston side being more athletic. And that goes back to my own understanding about how bias was used in athletics and used against Black people.” Others like Elias qualified such appraisals by saying playing style “would depend on the coach and the personnel … not necessarily the side of town that people are on,” but further asserted “at Lakecrest they still have a move-the-ball, you know, team-oriented style. Douglaston they have a little bit more of a, you know, freewheeling style.”

Play as structured versus non-structured—roughly akin to “coached” versus “street”—specifically merits attention, if not only for its racialized undercurrents, but also for its synchronicity with what facilities transition to formal play at higher levels [cf. (29)]. Former player Martin took Lakecrest's organization as a point of pride here when relaying his belief it advantaged players like him: “Lakecrest [is] like more of a collegiate level … The way that it was practiced over there … it was real deal … We were kind of a locked in team … When you watch Kentucky, they have a coach that like tells them what to do … We weren't just chucking shots.”

Invoking street versus structure directly, Kori paints it: “Lakecrest is very much so more of a structured style play. Historically, they run a lot more sets, there's a lot more ball screen actions, whereas Douglaston … were always just run and gun, more of the stereotypical just streetball type of play.” For Daran, “I think Douglaston kids obviously play outside a lot more. I think Douglaston kids [have], you know, obviously a more rugged [style].” Evan adds further on learning through outdoor play:

Douglaston are definitely like, scrappier … They're showboating and stuff, but it's … like, you're gonna get hit … people are gonna get hit, and like, just get used to it … It's more … traditional outdoor/ … /gritty [play]. You know, if there's blood, there's a foul. Otherwise, play through it, you know. Where a lot of the Lakecrest kids were not about that life. It's far more like “don't touch me.”

## What we share: (hurdles to) mentorship and facilitating play in contemporary Northland

Despite the potential depth of divides—both socio-economic or more objective on the one hand and corresponding subjective boundary work or appraisals of play on the other—we turn to what the Northland basketball community shares. This is their values, how they idealize Northland basketball, and what helps or hinders actualizing the youth basketball environment they best envision, particularly as challenges arise specific to Northland's weather, sports, and socio-demographic historical climates, as well as broad changes within the culture of youth sports just generally and the recent COVID-19 restrictions.

### Passing the torch

Consonant with pro-social value systems frequently held by enthusiasts (13), the Northland basketball community unsurprisingly idealized accessibility and ways basketball could foothold personal growth, produce well-rounded citizens, and facilitate mentorship of Northland's next generations. Reflecting access refrains, 41-year-old former Douglaston coach Katy simply asserted, “We were not very wealthy growing up … Basketball was a cheap sport, so I was able to do that.” Roman, a 66-year-old who played college basketball out of Lakecrest and remains involved in youth basketball, said likewise, “All you needed was a basketball [and] … tennis shoes … It's an inexpensive sport for kids” and, crucially, of community across Northland's divides. “The sport brings you together … It helps bond kids … If you’re growing up on one end of town … how do you know the other end of town? … This sport kind of brings you together.”

Many connected formative experiences with currently held organizational and mentorship roles. On learning interpersonal negotiation as a springboard (12–14), for Nick, “basketball … really helped me know how to interact and work with others in a cooperative way.” In addition, for the youth sports leadership program he works for now, “What we're trying to do to [is] equal the playing field for kids to have opportunities to be successful.” Regarding coaching (16, 40), Daniel described his pathway: “I … had some, some great coaches … In thinking about how they challenged me … I didn't have a leadership path other than through basketball … I often will think, like, ‘if not for basketball … what would I be doing?’/ … / I wanted to give back so I was coaching.” For examples of the ethos Northland coaches relayed to us, Katja said of her efforts, “We won't know if we're successful until 10–20 years from now. The goal is to teach them how to be better people through sport.” Complementarily, 73-year-old retired coach Cameron reflected on his life's work when saying that, at the youth level, “Your primary goal is not to win basketball games, but to help raise young people to be good, productive citizens and good people.” On informal mentorship, Evan told us “through basketball … I've helped a couple kids get their GED. I've helped a couple of kids get into college … I like using basketball to help kids figure out their drug issues, their mental health issues.”

### Hurdles

#### *“*Higher stakes” ball

Against best efforts, one area where consternations were overwhelmingly voiced came over movements toward greater financial investments being required to competitively cultivate youth talent, which was seen as contra basketball's longer-standing accessibility ethos and something that potentially deepened local inequalities. Bruno described the double-edge sword of the higher levels of personal training youth basketball aspirants increasingly receive, “I think there's some opportunities … like organizations that do training and stuff that like, [which] is great, but some kids just don't have the money to be able to afford … to get trained in basketball.”

However, more than anything, AAU basketball was locally named as an increasingly necessary ingredient for player ascension. In the following excerpt, Daniel not just notably attributes the new training norms Bruno references to be most closely embodied, not anywhere in Northland, but in the basketball program at Huntley, the wealthy suburban school in the Metropolis south of Northland, but he also well articulates the widely shared sentiments around AAU's emergent centrality:

The trends in basketball, where it is, it's leagues, it's travel team, it's AAU … Basketball is becoming more of a suburban sport … These wealthy suburban schools that are dominating high school basketball, it's like … when did that happen? And why is it happening? … Some of these kids, they have like personal trainers, and they're like developing various … sophisticated sport training techniques that are that previously had only been available to NBA players, and now they're like making it available to 16 year old kids in Huntley! … It's a shame … You very clearly see the social economic impacts of the way that we structure basketball now … There's this huge advantage to the very wealthy schools.

Locally, organizers felt that Douglaston youth who did not stand out as more elite players at early ages risked being further left behind because, among those without parents who can pay, external AAU sponsorships are commonly given first to young players who exhibit exceptional precocity. Here, Nick observes, “[I] see a more stark difference with Douglaston than I have probably ever before … I would say that socioeconomics has not provided opportunities for kids to maybe play … AAU basketball … The costs … have kept a lot of kids out.” From having previously coached AAU, Kori said, “When I was coaching AAU … we had like a $500 entry fee. And historically, people on the Douglaston side could not afford that whereas the people in Lakecrest could afford that.” More generally, Douglaston coach Elias underscored, “In the modern day basketball … the kids that become the best are the ones that are able to play AAU and offseason, which entails traveling and expense and parental support, you know, finances,” and of his approach to challenges he faces in growing his players:

I think a big thing … now living and coaching in Douglaston … is to find ways to develop basketball players, make ways for kids to play basketball … trying to find ways to kind of go back to the old days … before AAU … where we could develop basketball players and get them playing outside [instead] of having to have it be organized and expensive.

There are AAU opportunities in Northland. However, these are less developed relative to those available in the south Metropolis. Daran has been taking part in recent attempts to grow Northland AAU opportunities and expand sponsorships. In this excerpt, he describes his vision for what the future of Northland basketball could be with a more robust AAU presence and how it can aid basketball aspirants in contemporary times. He also crucially emphasizes the material limitations presented by what is seen as a lack of court space in Northland:

There's never ever, ever, ever been a good AAU program in this area that's geared towards making kids better and geared towards putting kids in a college, you know, playing college basketball … I think that's going to be a linchpin in like truly making this area a dominant basketball area. So AAU is going to be big. [But] then gym space. That's huge, man—why the hell do we not have a basketball facility here?

#### Facilities in Northland's climates

Playing space was almost comprehensively perceived as limiting Northland's potential. “You … don't have a lot of gyms available,” said Tony. Berndt adds plainly, “We need more gym space … in general in Northland.” Northland's weather is a major intersecting factor—to Janusz, “There's nowhere to play. It's cold at least eight months of the year here.” Elias turns this focus to development when saying, “It's always been hard to find a place to play in the winter … In terms of having a free, accessible, easy place for kids to go year round and play inside, or finding gym space in the winter for practices, it's not great.” The coach Katja noted the ironies of her players frequently not being able to practice indoors because of facilities closings due to outdoor conditions—“[if] schools close, we cancel … This year … I think they had six or seven days canceled from weather, and a lot of our facilities are those schools … Oddly enough, [this] indoor sport … [is dependent on] the outside weather.”

The “in season” limitations for basketball in Northland starkly contrasted with how hockey was viewed. In the words of lifelong Northland resident and former Athletic Director Katherine, “the height of basketball season is also the height of the cold season … You can't go to an outdoor rink.” In addition, Tony notes, there are “rinks every other block where kids could play hockey … Does weather plays a role in basketball? Yes. Our weather is conducive to hockey.”

In the city proper, outside the school gyms, there is currently only one fully publicly available court and two others that are run by non-profits and accessible with fees. Given the generally perceived difficulties with accommodating basketball during the sport's season, numerous Northland basketball community members voiced a need for, or dreamed of introducing, a new basketball facility to this mix. Looking across his life of involvement with Northland basketball, the 66-year-old homegrown Division 1 player Roman told us, “The biggest thing is … and I’ve tried … was trying to get a facility that's dedicated to basketball.” The 27-year-old Arvid, who grew through Northland basketball likewise to a Division 1 scholarship roughly four decades behind Roman, voiced similar sentiments when professing a facility, “Would help the basketball community in Northland. It would … bring more kids to get interested in it, and to bring more kids from the surrounding areas … to play in tournaments to playing games.”

In line with the comments of Roman, efforts to actualize additional winter facilities have been longstanding. Katherine provided context saying, “Northland desperately, desperately needs more facilities … We looked into economic feasibility and that really is difficult.” Complementarily, Janusz observes, “I don't think there's a lot of money going into basketball in Northland … If you want it to develop, you need to give it some tender loving care, and that involves money.”

As several specifically told us, the closest Northland has come to actualizing new winter facilities came with recent plans to potentially include basketball courts in a recently built sports complex, the Hartstone Center. However, as these informants generally saw it, because hockey dominates Northland and wealthier local families are more common in hockey, Hartstone was ultimately built without basketball. At present, it primarily houses hockey and soccer. Here, we receive Daran's account of the situation and how he believes it impacts his vision for a more robust local AAU circuit:

[Hartstone] was supposed to be earmarked for three basketball courts … It got scrapped because all the money's in hockey. If you go down to Hartstone, all those bricks have the names of donors, and like all have like a Hall of Fame for like anonymous donors. There are people giving like $10 million to this place, but they only wanted to use it for hockey/ … /There's no Hartstone for basketball … The hardest thing for us is like … next year, we’re gonna have 20–22 or 25 AAU teams. Where the fuck they gonna practice?

**Figure 1:**
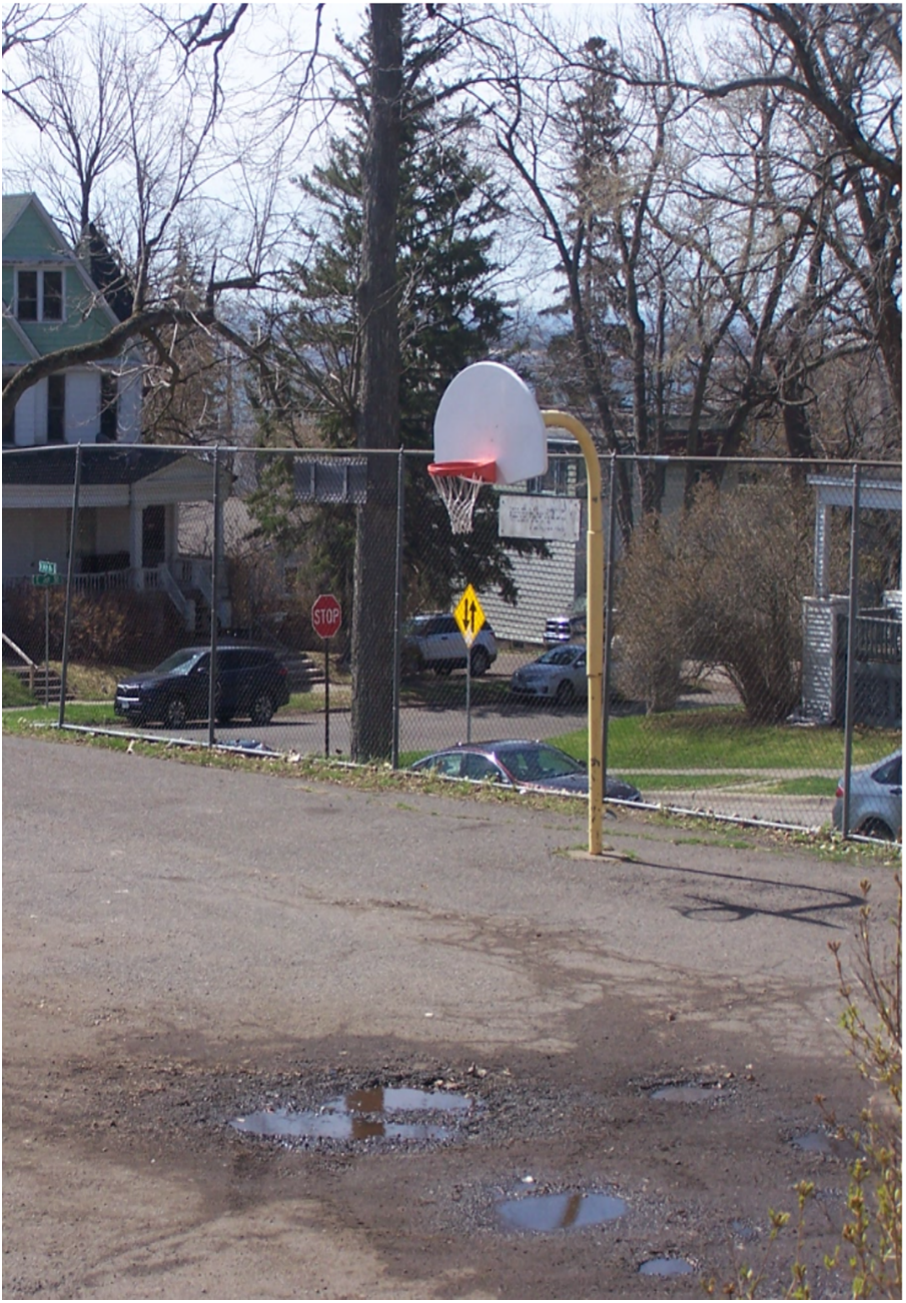
Northland summer hoops (June 2023).

**Figure 2:**
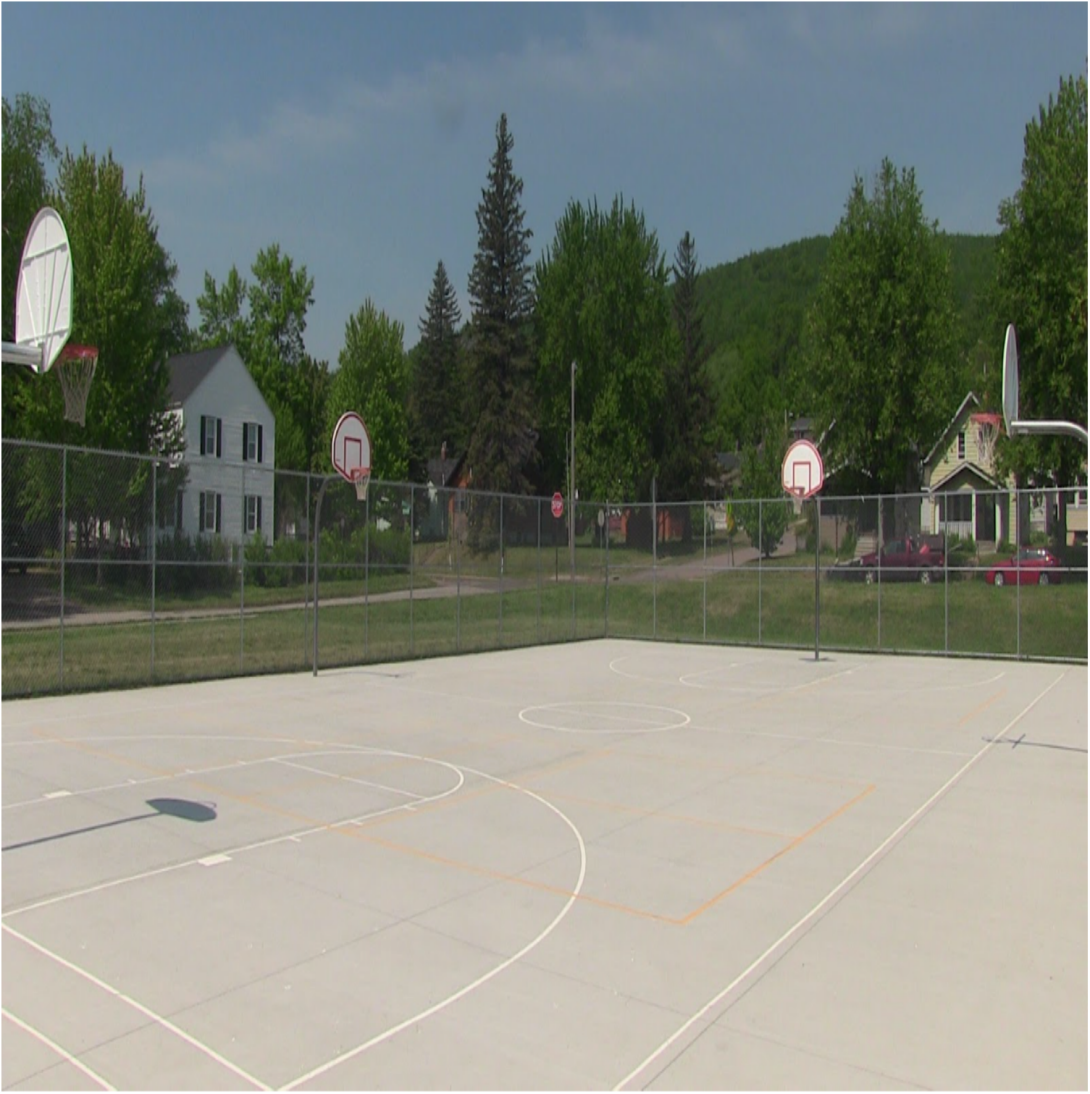
Northland summer hoops (June 2023).

**Figure 3:**
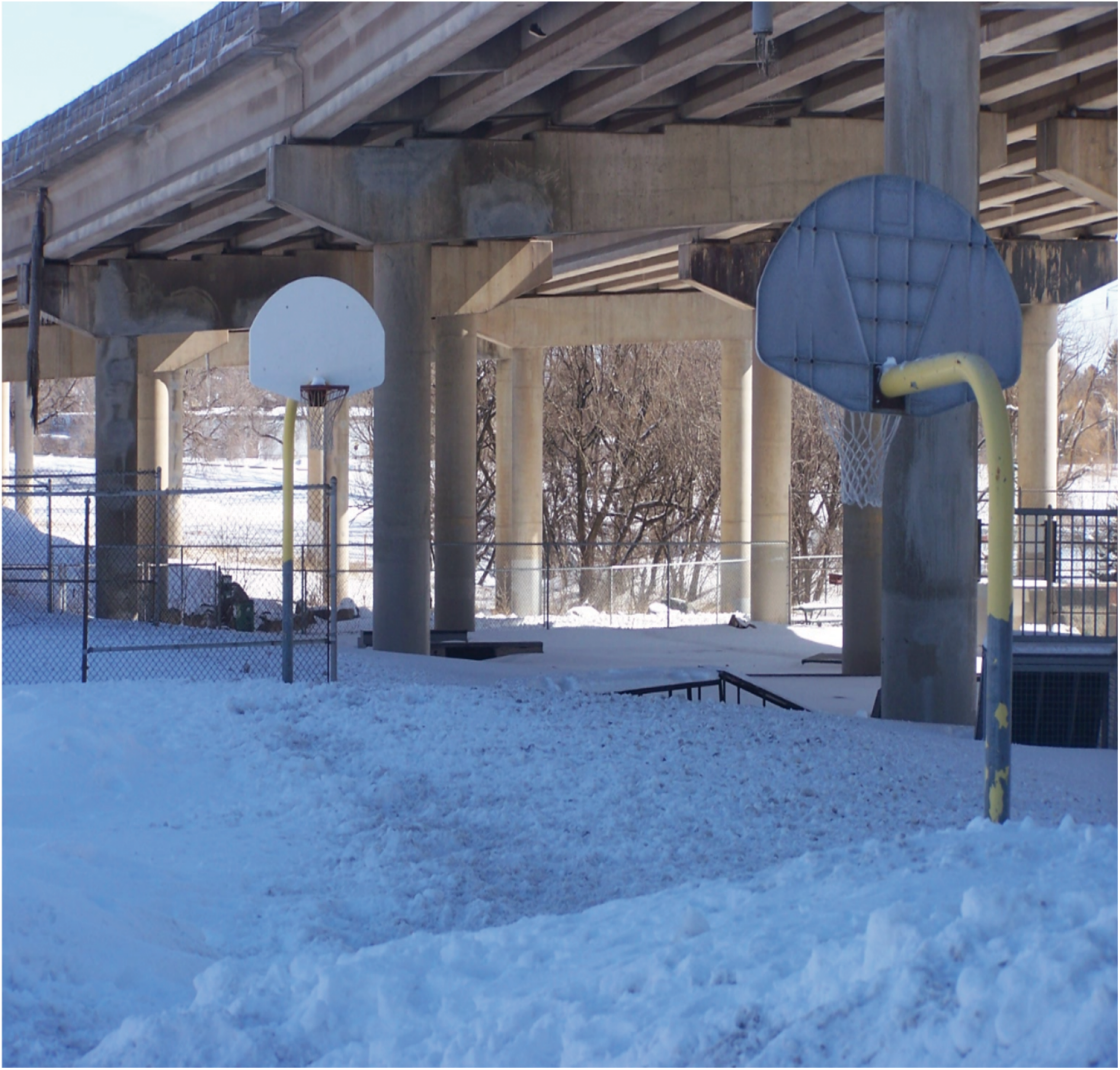
Northland winter hoops (January 2023).

**Figure 4:**
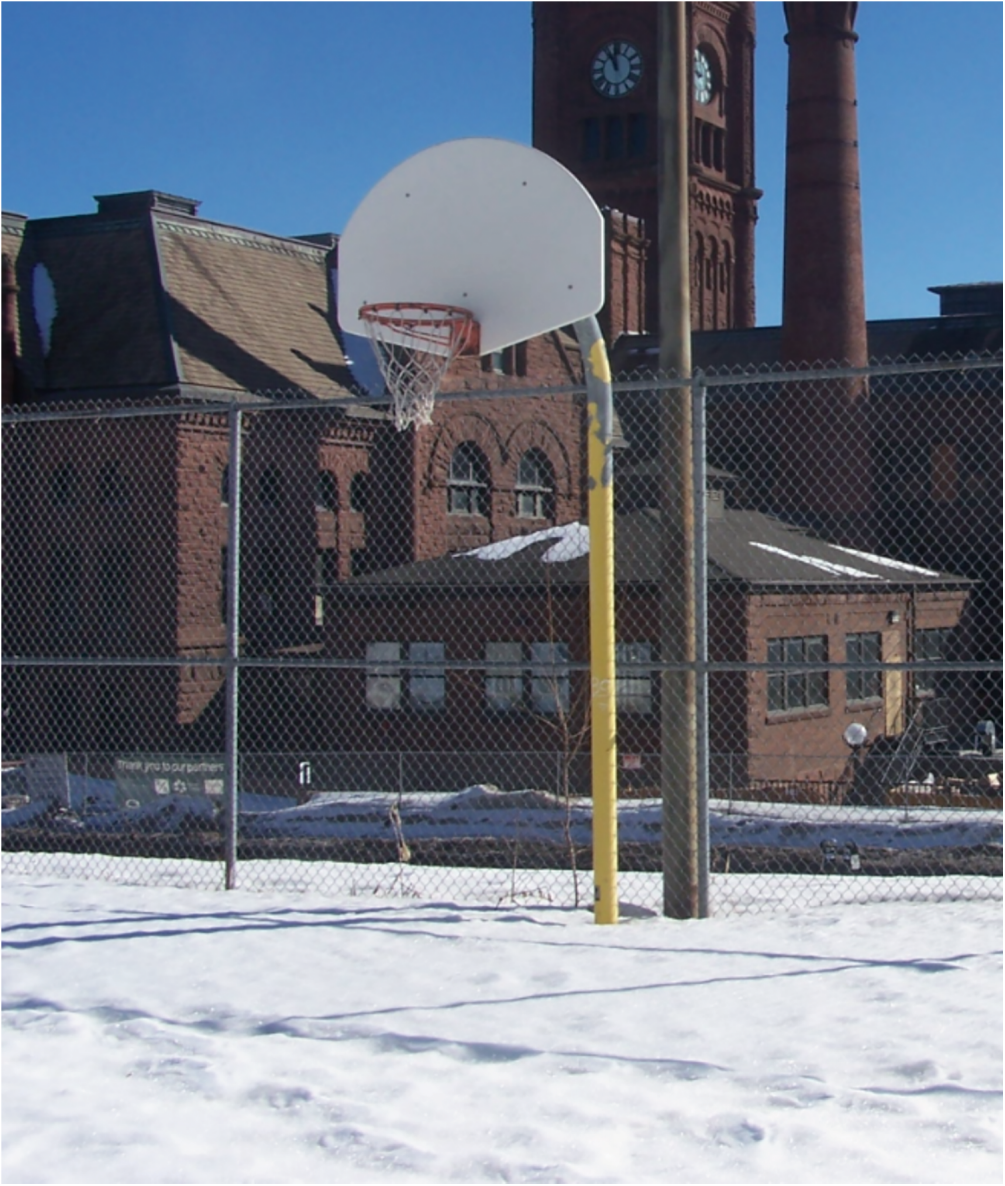
Northland winter hoops (April 2023).

**Figure 5:**
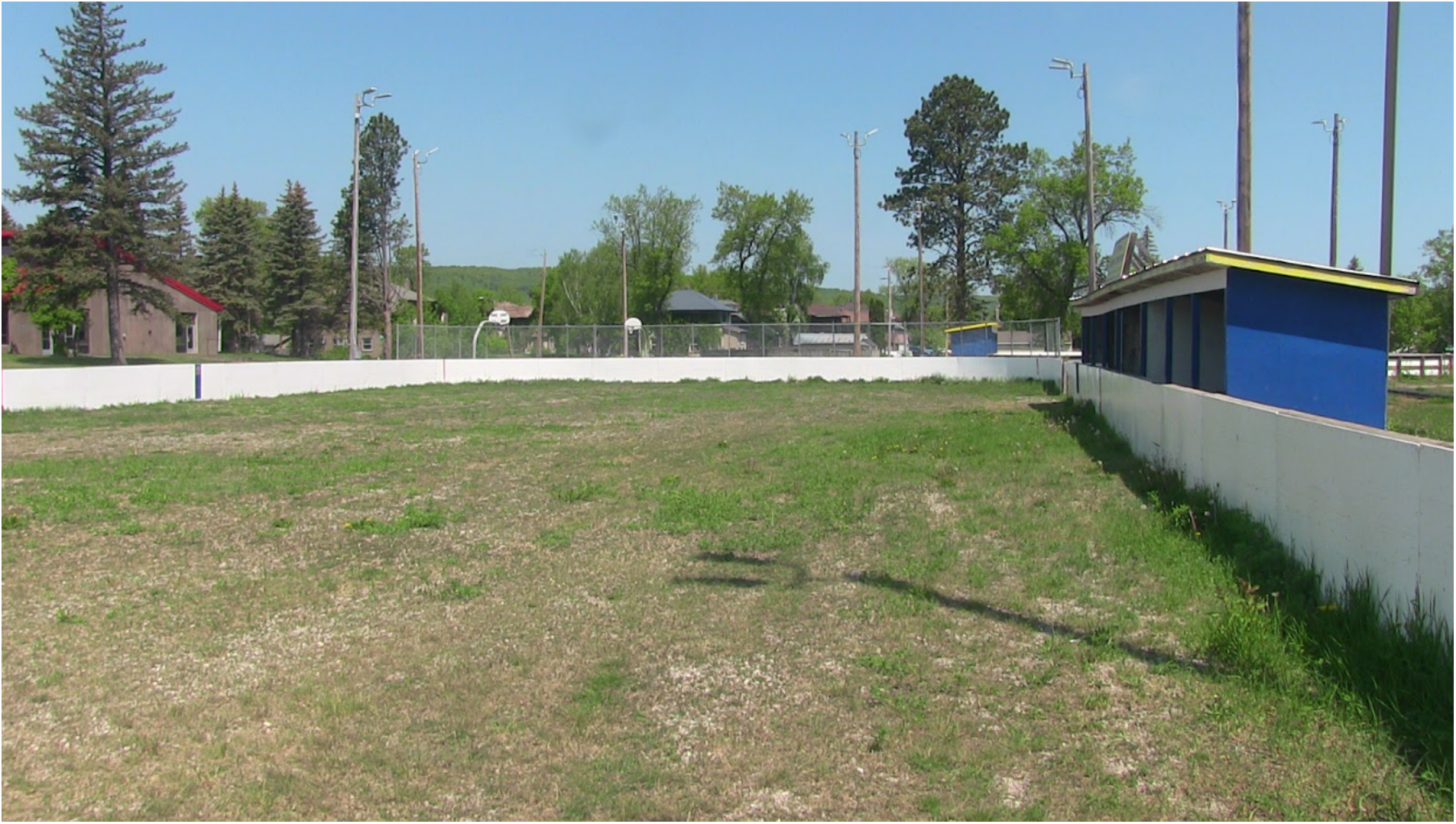
Rink with hoops in the distance (June 2023).

Regarding offseason play and practice, where the Hartstone Center provides hockey players an additional potential local option for summer ice time, numerous basketball hoops are available in Northland's public parks during warmer months (see Figures 1–5 for examples across seasons). Many fondly recounted time at these as youngsters. However, public courts or hoops may have their own specific difficulties with respect to funding and maintenance. In this vein, Daniel poignantly drew upon his political experience to describe local hesitancy for using public funds to revamp or build courts due to the limited number of months they can be used. He also discusses his perceptions that some opposition to full 5-on-5 courts in or adjacent to white neighborhoods specifically may be animated by fears surrounding the ways such courts are understood to serve as gathering centers and connect with “streetball” playing styles.

Outdoor court[s are] always a challenge in Northland, in part because the seasons are so short…From a public policies standpoint, they say, “well, you know, does it really make sense to build beautiful outdoor courts when it's really only going to be used for a couple of months of the year?” And then there was even for a time [the question of] “do we really want full court basketball, you know, in our neighborhood parks?” Or “do we just have half court?” … What are some of the factors in play there? … I think in some circumstances, neighbors pushing [against a] full court basketball court was kind of a racist act. You know, like … if we have full court basketball … it encourages a certain type of basketball that maybe we don’t want to have around.

#### Playing in and out of COVID-19

Summer outdoor play in Northland was seen as down relative to prior times. As Evan lamented, “There used to be really active … like three or four courts you could go to at any time … And now we could drive by them, and I never see people there.” Cameron likewise observed fewer homes with hoops: “You drive around … you don't see hoops up in the back yard anymore, like we always had … You'd see those all over the place in the old days.” Helga perceived what current play there was as split by neighborhood, “I do see kids playing in [old Middlehill]. I don't see them playing in [Lakecrest], ever … And I would notice because I used to live in Chicago. I lived next to some courts that were very active, 24/7.”

Our respondents to be sure voiced immense concern for Northland youths’ emergence from COVID-19 restrictions on several fronts (general education, socialization, and family relations), but we here discuss some of their perceptions when asked specifically about basketball. Public parks were restricted in Northland through much of 2020—“People couldn't get out, people couldn't do basketball,” as Quincy succinctly puts it. If these closures were not alone residually lessening public play into 2023, many perceived their diminished usage as reflecting aspects of contemporary youth basketball culture in ways that compounded with—and potentially negatively influenced player development through—COVID-19 restrictions. Much of this fell upon what was seen as an emphasis on gameplay practice over skill practice, or what can frequently be accomplished alone—e.g., fundamentals of dribbling, footwork, and shooting if a hoop is available. Reflecting many here, Tony appraised younger generations of players, “Kids don't want to practice anymore. They want to play.” Cameron noted regarding his perceptions of the current cultural structure of youth basketball, “Youth programs and all these youth tournaments … all they do is play games. So therefore the emphasis on playing games … They don't work on skills … Or I hear of [newer] coaches who just like to scrimmage.”

On receiving players back after restrictions, Kori correspondingly described, “Because of COVID … as far as basketball skills, I think they're way down from where they need to be.” Kristin more directly questioned whether her players undertook individual practice when stating, “I was coaching Douglaston during the pandemic … I know a lot of the kids that I happen to coach needed a lot of guidance … yeah, a lot of guidance … I don't know if they took initiative and responsibility to practice.” Elias connected this with the newer organizational norms of youth basketball:

I don't think COVID helped at all … because you just don't see many kids getting out there and practicing by themselves. You could have done that during COVID, but … I see very few kids doing that sort of thing … [Before COVID] the movement towards … trainers or coaches or organized … systems had already begun, was already in full force … I would say COVID furthered that.

Gameplay itself also suffered immensely. Age-specific developmental landmarks were noted. For Berndt, returning grade-schoolers lagged compared with prior cohorts in developing baseline bodily confidence: “The third … and fourth graders that had been in COVID … they just weren't as comfortable athletically … I put that on … COVID … You're … [supposed to] learn from your experiences. And when … that was limited … it definitely impacted our kids.” At the middle school level, when youth take on larger “team” or “game stage” concepts (58), Arvid noted, “Middle school … kids … that suffered the most … lost a year to development in terms of [playing] team-wise, how to play with a team how to play against others … The last two years worth of game reps [were lost] where that development was really important at that age.” Daniel, who also coached middle schoolers, more directly discussed the experiential ends of getting players back on track:

I was coaching … both before and after COVID … middle school basketball … It's challenging to coach middle schoolers …. [because] they're … going from the free for all … to a little bit more of a structured play … We lost the year with COVID, and … they didn't know how to interact with one another; they didn't know how to … feel like they were part of a team … It was only by the end of the of the season that you started to see [improvements] … For each grade, each age, they are missing a chunk.

Back to the high school level, Katja anecdotally referenced several newfound behavioral difficulties with players returning to organized activities: “I'm on my 11th year [coaching], and I have only sent someone home once because of behavioral issues, and that was last summer. And [we] had two [other] major incidents in the last two years.”

On the front of formal facilities, 34-year-old community college teacher and coach Tommy appraises, “In regards to Northland I think this is … one of those equity issues … during COVID [of] who had access to actually work on basketball skills … People that had money kept playing, and they made a way to do that, or they had trainers that they could keep going … I think that … just widened the gap between skills between the top and bottom.” More concretely here, Douglaston coach Kristin reported, “We were kind of fighting for gym time in early 2021.” She contrasted this with opportunities and amenities she believed Lakecrest players had, according to her subjective view, manifested in on-court performance once games resumed:

Well, during the pandemic … the students that went to Lakecrest had free gym time. They were able to play at Lakecrest whereas [at] Douglaston we couldn't play … I did feel a discrepancy when we played Lakecrest … They [also] had … new jerseys, everything, and they know [at] Douglaston we had to recycle them year after year … I know the kids felt it.

Finally, despite hardship and potential inequities, some points of optimism were noted for Northland basketball's possible future out of COVID-19. Consider Evan's account of a recent frustrating trip to Douglaston to give flyers for girls’ basketball that turned into perhaps his most successful recruitment effort ever:

I went over to Douglaston … to just hand out flyers to invite girls to come out. And I'll never forget … those kids all coming through the door in the morning all wearing masks … music going on in their ears, and nobody was talking … Everybody was just individual, and everybody looks sad, and everybody looked so down. And they would hardly look up [at me] to take this pink flyer … But what ended up happening was a lot of girls came out, like over 40 girls!/ … /A lot of [them had] not only … not played basketball, but hadn't played any sport. And a lot of them came up because they realized that … isolation … wasn’t good.

## Discussion

In his recent book *Our Kids*, Robert Putnam (59) painted a portraiture of a *de facto* class segregation forming across America in recent decades. Opposing “sides of the tracks” are more starkly demarcating varieties of towns and cities. Affluent children increasingly access disparately resourced education and more seamlessly engage the sort of extracurriculars that key success, often due to the deliberate efforts of their parents [see also (38, 39)]. By contrast, mentoring relationships otherwise vital to cushioning lower-income youth through their coming of age are more fractured and sporadic, and may correspond with declines in social trust, civic participation, and stability. Unselfconscious mixing across these lines—for example, by kids just playing together—is becoming lost in communities for Putnam, and this, to some degree, is scaffolding the widening of America's overlaying “opportunity gap.”

Across this article, we detailed the orientations, hopes, and subjective understandings held by members of the Northland basketball community, who, through the medium of basketball, voiced investment in providing the sort of mentorship and facilitating the connection for youth Putnam idealizes, all in a setting strikingly reminiscent of his broad-brush representations. The difficulties reported by informants for facilitating accessibility and equity across Northland's Lakecrest and Douglaston neighborhoods upon the splitting of Middlehill High School undoubtedly broadly reverberate here, especially when considered within the potentially individuating, higher stakes culture of contemporary youth sports broadly and through COVID-19 restrictions specifically. Regarding COVID-19, our data also notably begin to touch upon possible—general and age-specific—challenges surrounding player interest, development, and coaching, all of which underscore the regular attention that will need to be paid to these for years to come, particularly where some negative effects may be borne more by marginalized populations [cf. (60)].

Our larger approach of documenting Northland basketball in dialog with its historical climates also crucially foregrounds localized inflections that may even uniquely deepen or further splinter within such standard cleavages. There is a widely perceived dearth of facilities to prepare potential Northland basketball aspirants for higher levels of play as well as foster best-envisioned community wellness through basketball. Some of these challenges have to do with weather and the limitations it imposes in Northland. However, it may be more connected to basketball's secondary status to hockey. We are here reminded of Bourdieu's (61) take on the political economy of sports equipment, production, and administration, conceptualized as containing symbolic struggles over participation, funding, and defining general cultural dominance, as all this is requisitely shaped by various constellations of leisure time and certainly the economic and cultural capitals at play. His specific statement here not only pertains to ways general movements toward higher stakes training and organizational norms are arguably coming to define youth sports and condition corresponding social closures, but also significantly to intraregional sporting hierarchies like those which order Northland, in which facilities today are configured to such a state that offseason summer ice time may even be easier to secure for some youths pursuing hockey than gym space is available for winter basketball.

Deeper here, the cultural prioritization of hockey in this city containing historically embedded racial segregation nestled within a predominantly white larger region merits its own further pronounced note. Basketball significantly began to slip from any greater regional prominence it held through a calamitous economic downturn, and right as the predominant face of the sport shifted toward blackness in the 1970s and 1980s. Any sidelining of basketball across these years holds residual implications for contemporary Northland youth who might wish to engage in the sport because of cultural orientation and esthetic taste or who are just priced out of sports like hockey. While this can potentially animate processes like “placemaking” among underserved and racialized youth within Northland's rooted residential and racial inequities (22), its current potential negative consequences for perceived suitability for higher levels of formal play may be exacerbated in times that increasingly advantage middle- and upper-class youth possessing the resources to meet the investment and formal training requirements of higher stakes youth sports (cf. (62)).

Likewise, as per the narratives of numerous among our white respondents specifically, any barriers to or de-emphases on basketball may additionally come with a limiting of the racial awareness (including cultural whiteness) they believed was cultivated through the interracial touchpoint that playing basketball specifically offers in a place like Northland. In a larger historical light, any closing of effective white engagement with racial learning through basketball in this local context is borne from not only the sport's cultural deprioritization across recent decades, but also the more direct and concretized structural disinvestment in racial and socio-economic diversity that came with the shuttering of Middlehill High. In this more starkly bifurcated post-Middlehill landscape, insofar as black or non-white youth would be less likely to learn about racial dynamics or their communities by engaging in a white-coded sport like hockey (compared with white kids playing the more black-coded basketball in this context), any lost socio-political potential of basketball as a vehicle for social inclusion underscores the potential importance of focusing so closely on a specific sport like basketball in tandem with its deeper local inflection points.

Still, we must again emphasize here that the perspectives foregrounding this account only reflect various positions and perspectives in—and around—Northland basketball. Although the enclosed reports are, therefore, certainly not objective, definitive, or similarly applicable to other localities in full (nor necessarily reflective of how Northland hockey stakeholders might see things), the basketball community's starker descriptions from within Northland's specific weather, sport, and racial–political climates may prove clarifying for approaching issues of organizational structure, facilities access, and mentorship in other localities that are likewise broadly colder, whiter, and laden with socio-economic inequalities, or in which various sporting hierarchies pervade.

Regardless of where they are working, we are also compelled to underscore that Northland coaches and organizers are passionate about basketball and the development of their players on and off the court, across the whole region, and in both remaining city schools. The Northland youth basketball community embodies Hollander's (13) belief in basketball as a social institution that can key better worlds. Here, we speculate, as our informants no doubt do, whether a more expansive youth basketball presence in Northland could come to serve as an effective general community builder and deliberate instrument for actualizing local multicultural social policy in ways akin to his account of basketball and immigration in Toronto in *How Basketball Can Save the World* (13).

Such policy and certain mentorship questions generally begged by all this point to tensions running through this article that certainly reside beyond its scope of adjudication, but that compel direct consideration ahead from youth sport community members as well as scholars and policy-makers. The organizers, coaches, and former players of varying profiles we spoke with resoundingly believed that strong community connection was vital to their personal growth and, where applicable, progression to higher levels of basketball. However, in the present landscape, the question arises of at what point is the community ethos of basketball—or any other sport idealized to be widely accessible and playable with limited resources, like soccer—betrayed if those in supervisory roles must increasingly aim toward building in-roads to, or even just mimic, the individualizing and privatizing currents of the youth sports industry. Further here, and by contrast, where are youth best served by more informal or self-directed play as the 21st century deepens, to what ends, and with whom? If successful player development pipelines are increasingly residing in wealthier suburban settings and requiring higher individualized investment thresholds, our work here only underscores the pronounced attention that needs to be paid to the degree to which higher echelons of play may be becoming reserved for middle classes with access to formal facility and training spaces. Correspondingly, the question of the extent to which still culturally predominant images of uplift and mobility—through things like “street play”—are becoming less a thing connected to viable reality than a marketing trope in sports like basketball looms large. For basketball specifically, this is especially so in the face of the ways post-2008 public park divestments, gentrification, and, more recently, COVID-19 have all accelerated outdoor court closures across America.

Our documentation additionally underscores that a decidedly grassroots level of analysis is also just a needed voice if we are to most fully understand how feeder systems shape in today's higher stakes environments for youth sports, which marks somewhat of a contrast to the frequent focus falling on the pro and collegiate game, elite players possessing the highest of prospects, or new middle-class parentage cultures and how these connect with the demands of keeping up with the mushrooming youth sports industry. The ways cultural and social capitals shape boundary orientations to—and condition possible dangers for—engaging differential opportunities that might be available across places like Northland's Douglaston versus Lakecrest city school sports programs should certainly reverberate in other racially and class-stratifying contexts. In general, the issues of adequate facilities and surrounding mentorship questions foregrounded by this article's community account that have so long been central for Northland basketball specifically are likely to be broadly informative where and insofar the play of youth sports indeed becomes more rigidly organized, higher stakes and requiring of more movement indoors.

To us, on all fronts, adequate and available (in- and out-season) facilities are the obvious ideal or minimum baseline ingredient needed for beginning to meaningfully address any of these issues. Yet, in the case of Northland, given its environmental and historical–cultural context, it remains unsurprising that our story gleaned from the basketball community highlights the ascendancy hockey enjoys and the ways hockey orders the political economy of Northland sports. Inside Northland and out then, how can larger inter (and intra) sport cultures of sharing and cooperation be forged within the racial, socio-demographic, and cultural dynamics that shape local sporting hierarchies of any type, especially through times ahead that appear likely to evermore be individually focused and industrialized, if not outright shaped by private donation structures? What does seem clear is that casting the deeper entanglements of all such issues into their fullest light will require mindful connection with on-the-ground organizers invested in actualizing thriving youth sports cultures in their specific communities, something which we can only hope we have modeled with this grassroots account of Northland basketball.

We note in closing that numerous other high schools and adjacent locations are part of Northland regional basketball, and other sports (e.g., soccer and football) comprise a significant part of Northland youth sporting culture. The tale of these three city schools and the local dynamics between hockey and basketball so strongly anchored sampled informants’ understandings of how local youth basketball was conditioned, however, they could not but become this document's central focus. Douglaston's programs experienced some of their worst seasons on record the year following COVID-19 restrictions. As of this writing, the Douglaston boys joined Lakecrest above 0.500 the past season, and the Douglaston girls were able to improve their mark.

## Data Availability

The datasets presented in this article are not readily available. Requests to access the datasets should be directed to tork0032@d.umn.edu.
